# Performance Assessment of Low-Cost Thermal Cameras for Medical Applications

**DOI:** 10.3390/s20051321

**Published:** 2020-02-28

**Authors:** Enrique Villa, Natalia Arteaga-Marrero, Juan Ruiz-Alzola

**Affiliations:** 1 IACTEC Medical Technology Group, Instituto de Astrofísica de Canarias (IAC), c\Álvaro Martín Díaz 2, 38320 La Laguna, Tenerife, Spain; narteaga@iac.es (N.A.-M.); Juan.Ruiz@ulpgc.es (J.R.-A.); 2 Departamento de Señales y Comunicaciones, Instituto Universitario de Investigación Biomédica y Sanitaria (IUIBS), Universidad de Las Palmas de Gran Canaria, c\Paseo Blas Cabrera Felipe “Físico” s/n, 35016 Las Palmas de Gran Canaria, Las Palmas, Spain

**Keywords:** thermal cameras, low-cost, medical applications, diabetic foot, microbolometer, NETD, non-uniformity

## Abstract

Thermal imaging is a promising technology in the medical field. Recent developments in low-cost infrared (IR) sensors, compatible with smartphones, provide competitive advantages for home-monitoring applications. However, these sensors present reduced capabilities compared to more expensive high-end devices. In this work, the characterization of thermal cameras is described and carried out. This characterization includes non-uniformity (NU) effects and correction as well as the thermal cameras’ dependence on room temperature, noise-equivalent temperature difference (NETD), and response curve stability with temperature. Results show that low-cost thermal cameras offer good performance, especially when used in temperature-controlled environments, providing evidence of the suitability of such sensors for medical applications, particularly in the assessment of diabetic foot ulcers on which we focused this study.

## 1. Introduction

Thermal infrared sensing technology has experienced much development over the last decades due to a new generation of uncooled infrared (IR) thermal sensors based on microbolometers [1]. These detectors use microelectromechanical system (MEMS) techniques for manufacturing large 2D arrays, reducing the cost while offering a high sensitivity and good image quality [2]. The popularity and applicability of these sensors in multiple fields, both civilian and military, have contributed to the improvement of this technology in terms of noise as well as cost reduction and increased detector size.

Thermal imaging technology has raised significant interest in the medical field, since skin temperature variations can be related to different medical complications such as inflammation, ischemia, or infection [3,4,5]. Thermal imaging provides temperature functional observation as well as tissue structural thermal properties [6]. Applications based on thermography have been proposed for the monitoring and assessment of a diverse range of pathologies including dermatological complications, tumors, diabetic neuropathy, arthritis, vascular disorders, and other circulatory disturbances such as Raynaud’s phenomenon [7,8,9,10,11,12,13]. Furthermore, several applications have employed active thermography in which temperature is measured in dynamic states resulting from thermal provocation (heating or cooling) [14,15,16]. In addition, a number of e-health applications have proposed the use of IR thermography [9,17]. In the case of a diabetic foot, a database already exists to promote early diagnosis for this pathology [18]. The possibility of uploading self-monitoring images to a database has a twofold benefit. First, the subject gets a report if a warning sign is found, and then a publicly available database increases, improving the efficiency of the early diagnosis. Nevertheless, despite the increasing popularity of such applications, clinical thermography has not yet become a commonly used technology. This can be partially explained by the fact that, in most of the proposed applications, the selected sensors are still relatively expensive, bulky, and difficult to handle for patient self-monitoring. 

In the last few years, some manufacturers have presented new low-cost, off-the-shelf infrared apparatuses based on microbolometers including the optics and electronics, which are compatible with mobile devices such as phones or tablets. These sensors open a new venue for medical infrared imaging, as they present a competitive potential for home-monitoring applications, facilitating the accessibility and usability of this technology. However, these low-cost models offer reduced capabilities as compared to high-end devices mainly due to the sensor spatial resolution, simpler optics, housing, and electronics.

To the authors’ knowledge, the viability of using low-cost sensors in clinical applications, instead of the high-end microbolometers, as well as the impact of their performance have not been previously studied in detail. Specific low-cost microbolometers have been characterized without comparing them to scientific or industrial-class models nor analyzing the impact of the performance reduction in specific applications [19]. Long-wavelength infrared (LWIR) low-cost cameras have often been analyzed based on the data provided by the manufacturer and a visual inspection of the acquired images [20,21]. Furthermore, specific low-cost LWIR detectors have been characterized focusing on their applicability in areas not related to clinical pathology assessment or monitoring [22,23].

This paper aims to characterize thermal sensors and analyze their feasibility for medical applications, particularly the assessment of diabetic foot anomalies. Several studies have been performed to test the viability of infrared imaging on diabetic complications based on the study of temperatures of the sole of the foot [24,25,26,27]. Before an ulcer is visible, a previous subcutaneous anomaly may cause a local temperature variation on the skin that can be detected with thermal infrared sensors. The assessment procedures can be typically classified into one of the following main groups: independent limb temperature analysis, contralateral temperature symmetry analysis, temperature distribution analysis, and external stress analysis [28]. All these methods require a thermal imager able to provide a medium or good spatial resolution, repeatability and reproducibility of the measurements under different conditions, low noise, and low fixed-pattern noise (FPN). Traditionally, technical requirements for thermographic applications for ulceration risk detection are fulfilled by medium or high-end cameras [9,10,11,29]. Therefore, the main objective of this work was to characterize low-cost microbolometers and compare their performance to a reference high-end thermal camera. These sensors were characterized in terms of non-uniformity (NU) effects together with their dependences on ambient temperature, noise-equivalent temperature difference (NETD), as well as camera response. 

## 2. Materials and Methods

This section explains the infrared cameras’ selection and their analysis, comparing the manufacturer-provided characteristics with the measured ones, after a comprehensive mathematical and physical description of the parameters. 

### 2.1. Infrared Cameras

#### 2.1.1. Low-Cost and Smartphone-Compatible Infrared Cameras

Several commercially available infrared cameras were evaluated for self-monitoring medical applications. The technical requirements included low-cost, medium or high detector size, low weight, built-in optics and electronics, in addition to compatibility with mobile devices. Four cameras fulfilling these requirements were identified and their characteristics, as provided by the manufacturers, are summarized in Table 1.

**Figure 1:**
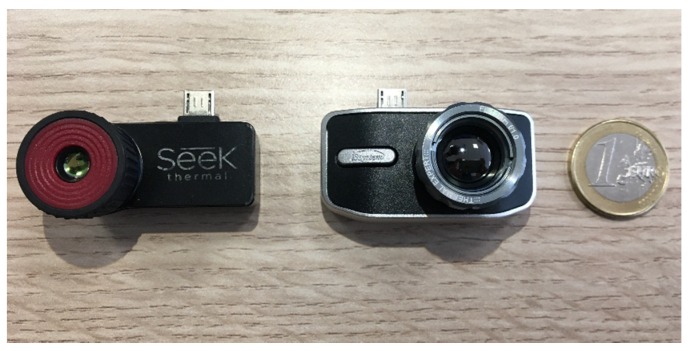
Selected thermal cameras: ST (left) and TE-Q1 (right).

The Flir camera was discarded because of its small detector area, while the Opgal camera was not considered due to its higher cost, which was roughly double the other models. A Seek Thermal CompactPRO (ST) and two units of Thermal Expert TE-Q1 Plus (TE-Q1) cameras (see Figure 1) were chosen and characterized.

#### 2.1.2. Reference, High-End Thermal Camera

An INO IRXCAM-640 scientific-class thermal camera based on a microbolometer was used for the tests. This camera was manufactured by the Canadian corporation INO (National Optics Institute) and based on a 640 × 480 pixels ULIS uncooled microbolometer model ULIS-04-17-1 [30]. The sensor is considered uncooled because no cryogenic system is used to reduce its temperature. However, a thermoelectric cooler (TEC) is integrated to stabilize the temperature as in many high-end IR sensors. The INO IRXCAM-640 camera includes a JANOS lens, optimized for the thermal infrared range (Surnia model, f/0.86, 95% typical transmission in the LWIR range). This system offers high-quality features such as low-noise electronics, 16-bit converter, and two parallel readout circuits, as well as fast optics that maximize energy transmission to the sensor surface. 

**Figure 2:**
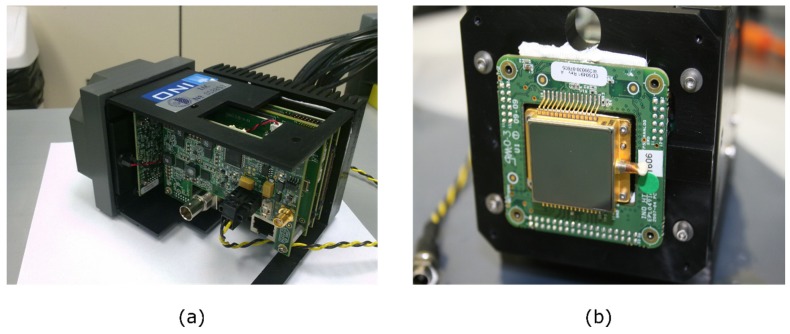
High-end thermal Camera: (**a**) INO IRXCAM-640 housing and electronics; (**b**) Microbolometer (ULIS-04-17-1) mounted on the housing.

This camera has been previously used for prototyping and testing purposes in astrophysics instrumentation [31]. However, it has to be mentioned that new microbolometers with 1024 × 960 pixels already exist in the market. Figure 2 displays the INO IRXCAM-640 and Table 2 summarizes its characteristics.

### 2.2. Experimental Setup

**Figure 3:**
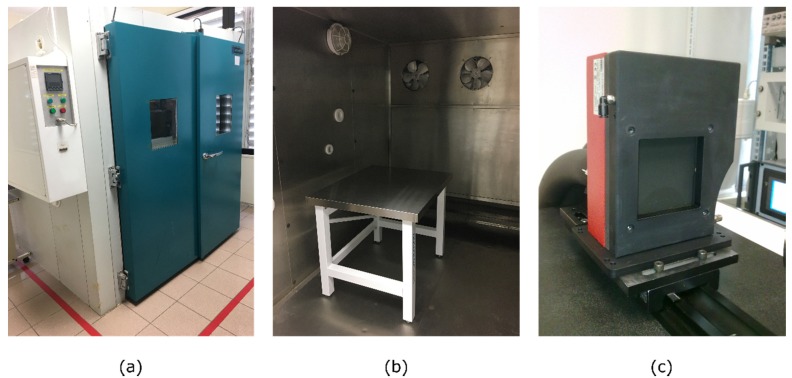
Laboratory facilities: (**a**) Dycometal WALK-IN MOD entrance door; (**b**) Workbench inside the climatic chamber; (**c**) DCN1000-L3 laboratory blackbody.

The experimental setup used for the cameras’ characterization is shown in Figure 3 and included the following:

- Climatic chamber WALK-IN MOD CCM20/8000 (Dycometal) employed to control the ambient conditions regarding temperature and humidity
- Infrared radiance source DCN1000-L3 (HGH Systemes Infrarouges) which provides an emissive area size of 75 × 75 mm^2^ and a temperature range from −40 °C to 150 °C. This blackbody head provides a temperature-controlled emitting dark surface (emissivity 0.98 ± 0.02) with a thermal uniformity lower than 10 mK root mean square (*rms*) at ambient temperature (±5 °C) and an absolute temperature accuracy lower than 50 mK *rms*.

### 2.3. Test Description

#### 2.3.1. Detector Response Model

A simplified detector response model was considered, which is dependent on the irradiance at every focal plane array (FPA) pixel, including the non-uniformity (NU) effects. This irradiance is a function of the object temperature ($T_{\mathrm{obj}}$) and its emissivity (ε), the background temperature ($T_{\mathrm{amb}}$), the wavelength range (Δ*γ*), and the optical system. Therefore, the simplified response of each pixel can be mathematically expressed as follows:

(1) $$R_{i,j}\left(T_{\mathrm{obj}},\;T_{\mathrm{amb}},\;\Delta \gamma ,\varepsilon \right)=G_{i,j}\cdot Y_{i,j}\left(T_{\mathrm{obj}},\;T_{\mathrm{amb}},\;\Delta \gamma ,\;\varepsilon \right)+B_{i,j}+n_{i,j}$$

where *G_i,j_* and *B_i,j_* are the gain and bias parameters, respectively, modeling FPA NUs. n*_i,j_* represents the pixel readout noise and follows a Gaussian distribution. The emissivity of the skin is high (ε = 98.9 ± 1%) and close to that of a blackbody [32,33], thus reflectivity is negligible. In those cases in which the object is close to the camera, the atmospheric transmission effects can also be neglected. Similarly, when the object temperature range is small, as in medical applications, and according to Planck’s Law [34], the irradiance dependence on the object temperature can be simplified and considered linear. However, the effects caused by the lenses could be significant, creating NUs in the irradiance at the FPA. In this work, any NU effect created by the lens was included in the FPN of the microbolometer.

#### 2.3.2. General Functioning Validation

The available software development kits (SDKs) provided by the manufacturers to control and read the cameras were studied to detect user-transparent image processing, which might influence the subsequent tests. In this work, all the in-house data processing was performed in MATLAB (R2010b).

Additionally, sample images were acquired with each camera in order to offer a visual comparative. During this process, defective pixels were noticed and excluded from the subsequent data analysis. Furthermore, all cameras were stabilized for a period of approximately 15 min prior to image acquisition. This period was selected because it is required to stabilize the infrared sensor, reducing the errors due to the heating of the internal electronics. Furthermore, the same period is recommended by thermography guidelines to stabilize the temperature of the skin [35,36,37].

#### 2.3.3. Noise-Equivalent Temperature Difference

The NETD is a figure of merit for infrared sensors which represents the temperature change that produces an output signal equivalent to the rms noise [31,38,39,40,41]. Thus, this value defines the thermal resolution, that is, the minimum resolvable temperature increment, which can be expressed as follows [34]:

(2) $$\mathrm{NETD}=V_{n\;}\frac{\Delta T}{\Delta V_{s}}$$

where V_n_ is the noise signal, ΔT is the temperature variation, and ΔV_s_ is the signal value corresponding to ΔT sensed by the detector. The noise signal is measured as the standard deviation value of the sensor pixels when a homogeneous image is expected. It has to be remarked that constant non-uniformity effects in the sensor and irradiance noise should not be usually included in the NETD estimation.

Since the NETD value is an important feature in microbolometers, it is normally provided by the manufacturer. However, different methodologies are commonly employed that prevent a proper comparison between cameras. Therefore, the same setup and methodology were used in all performed tests to ensure comparable results. In order to compute the spatial noise without including unwanted non-uniformity effects, an accurate procedure previously used at our laboratory for scientific tests was employed [31]. This procedure consisted of acquiring a series of one hundred consecutive images and, subsequently, subtracting consecutive images pairwise, thereby offering ninety-nine differential images and ensuring the removal of the constant non-uniformity. Afterwards, the noise standard deviation for each differential image was computed as the standard deviation in the pixel values of the image divided by √2 to compensate for the image subtraction. Finally, the noise signal (*V_n_* = $\sigma_{n_{i,j}}{}^{2}$) was represented by the mean of the noise standard deviation computed for the set of images. In order to convert images from raw data into temperature, the $\Delta T/\Delta V_{s}$ ratio was calculated by fitting the imaging model (Equation (1)) as the average value of the *G_i,j_* matrix (described below in Section 2.3.4). 

Regarding the TE-Q1 camera, the same conversion was applied to ensure that the factory settings did not compromise the results. In addition, the SDK provided by the manufacturer appeared to include some image processing during the acquisition. Thus, a custom-made application, based on the manufacturer’s SDK, was employed for the acquisition. Furthermore, this camera presents a significant quantization interval when compared to the sensor noise signal, which might influence the measured NETD value. This effect was taken into account by computing the spatial noise as the square root of the sum of two squared errors, the quantization and the estimated sensor signal errors. The latter was estimated using a Gaussian function, which fits the measured noise histogram. 

Images were acquired inside the climatic chamber at 25 °C (temperature variation less than ±0.2 °C) and the blackbody was stabilized at 27 °C (300 K). The distance of the cameras to the blackbody was selected so that the blackbody emissive surface completely filled the infrared sensors’ field of view. Since each camera has its own lens, their f-numbers differed accordingly. However, this was not taken into account in the calculation of the NETD. Table 3 summarizes the NETD tests carried out for each camera.

#### 2.3.4. Non-Uniformity Correction and Residual Non-Uniformity 

The performance of infrared thermal detectors is known to be strongly affected by the FPN or NU in the spatial response of the microbolometer array [42,43,44]. Individual elements in the microbolometer FPA differ in responsivity to incoming irradiance, causing non-uniformity effects. On the other hand, they offer a good linearity with the incoming photon flux and pixel irradiance. Assuming this linear responsivity, the NU effects in the FPA can be modelled by spatially varying gains and offsets (or biases) for every pixel [44]. 

The FPN and NU, mathematically represented by *G_i,j_* and *B_i,j_* in Eqution (1), are unwanted characteristics of any infrared camera. If the observed object has a homogenous surface temperature, a perfect thermographic camera should offer the same response for all the pixels in the image. Usually, thermal systems use NU correction (NUC) algorithms. These algorithms are used to estimate *G_i,j_* and *B_i,j_,* and, subsequently, apply the required correction, based on these values, to remove the NU.

The traditional NUC techniques include the one-point and two-point NUCs [45,46]. The one-point NUC is the simplest algorithm which uses only one temperature point and, accordingly, it can only estimate the *B_i,j_* matrix. This method is commonly used to update the NUC using a nearly constant temperature shutter in the camera or manually blocking the incoming radiation with a homogeneous temperature object. The two-point NUC provides an estimation for *G_i,j_* and *B_i,j_* using two temperature points. In this case, a reference object, preferably a laboratory blackbody, is employed and set at two different temperatures. A more accurate estimation can be obtained using multiple temperature points of the reference object and a least squares estimator (LSE) to compute *G_i,j_* and *B_i,j_*.

In this work, NUC was performed in a controlled environment, that is, the climatic chamber’s temperature was set at 25 °C. An LSE with nine blackbody (*T_obj_*) temperature points was employed, ranging from 10 °C to 50 °C in 5 °C steps. A set of sixteen images for each temperature point were acquired following the previously described procedure [31,47]. Once *G_i,j_* and *B_i,j_* are computed, the corrected image, $X_{i,j},$ and its mean value are defined as follows:

(3) $$X_{i,j}=\left(\frac{R_{i,j}\left(T_{obj}\right)}{G_{i,j}^{25\;{}^{\circ}C}}-B_{i,j}^{25\;{}^{\circ}C}\right)$$

(4) $$\bar{X}=\bar{\left(\frac{R_{i,j}\left(T_{obj}\right)}{G_{i,j}^{25\;{}^{\circ}C}}-B^{25\;{}^{\circ}C}\right)}$$

Furthermore, the quality of the correction provided by the same NUC algorithm can vary according to the respective camera characteristics, such as the noise, the stability of the measurements and other features included in the cameras as internal shutters (ST and INO IRXCAM-640) or TECs to stabilize the FPA temperature (INO IRXCAM-640). In order to validate the quality of the performed NUCs, the residual non-uniformity (RNU) was estimated as described in [31,48]:

(5) $$RNU=\frac{1}{\bar{X}}\sqrt{\frac{1}{M\cdot N}\overset{M}{\underset{i=1}{\sum }}\overset{N}{\underset{j=1}{\sum }}{\left(X_{i,j}-\bar{X}\right)}^{2}}$$

where *M* and *N* represent the total number of rows and columns in the image, respectively. These values vary accordingly for each tested camera. The RNU was computed by setting the blackbody temperature, *T_obj_*, to 32 °C. This temperature differed from those used to estimate the NUC parameters. A set of 100 consecutive images was averaged in order to reduce the temporal noise from the validation image. 

In addition, a more intuitive value of the residual NU was estimated by the NU error e_NU,_ expressed in temperature units and computed as follows:

(6) $$e_{NU}=\sqrt{\frac{1}{M\cdot N}\overset{M}{\underset{i=1}{\sum }}\overset{N}{\underset{j=1}{\sum }}{\left(X_{i,j}-\bar{X}\right)}^{2}}$$

The peak-to-valley error (e_P-V_) was also calculated. The SDKs of each camera already perform a defective pixel correction. However, after applying the NUC, some defective isolated pixels were detected by visual inspection. These outlier pixels, such as hot or cold ones, were excluded from the e_P-V_ computation by discarding twenty pixels, ten with the highest values as well as ten with the lowest ones.

#### 2.3.5. NUC Validity Under Varying Ambient Conditions

NU effects in microbolometers depend often on the room temperature. Temperature variations in the optics or objects near the sensor, including the camera case, modify the level and distribution of unwanted irradiation in the focal plane, and temperature variations in the FPA influence its responsivity. Accordingly, the NUC providing the optimal correction at a certain room temperature is not optimal when this temperature differs. This effect can also be considered as a temporal drift in the camera calibration parameters [49], since the ambient temperature varies with time in non-controlled environments. 

The selected cameras already include methods to minimize the effect of room temperature variations. The ST camera includes an internal shutter that automatically performs a one-point NUC correction when internal thermal variations are detected. In addition, this camera is equipped with an FPA temperature sensor. The TE-Q1 camera also provides an internal FPA temperature sensor. Moreover, as previously mentioned, the INO IRXCAM-640 includes a TEC to stabilize the FPA temperature avoiding NUs related to thermal spatial gradients or time variations in the sensor. Additionally, this camera includes a shutter mechanism, which combined with an external blackbody offers a periodic bias reference to improve the image quality. Therefore, this setting was enabled to maximize the INO IRXCAM-640 performance in the tests, since this camera was considered the reference in terms of quality. 

To study the importance of room temperature variations in the selected cameras, the previously described RNU, e_NU_, and e_P-V_ tests were repeated setting the climatic chamber at 10 °C and 40 °C as room temperatures. These temperature values were chosen because they approximately represent the temperature limits expected for the desired application.

#### 2.3.6. Detector Response Curves with Varying Room Temperature

The temporal stability of the camera response is an important feature in medical applications in which longitudinal studies are required. In these studies, repeated measurements of the same variables at different temporal points (short or long periods of time) are mandatory. Anomaly detection in the temperature pattern of a patient requires a longitudinal study, and thus the acquired images should be comparable. However, the microbolometers’ response and NU vary considerably unless the system is under controlled ambient conditions, which is not always feasible. These accuracy problems are mainly caused by room temperature variations, which influence the temperature of the FPA, the optics, or objects near the sensor. Unlike NU, which represents relative differences in the FPA, the response curve variation represents a global variation in the camera. Therefore, it can be considered an alteration in the temperature calibration of the camera.

An exhaustive characterization of these response curves of each tested camera at varying room temperatures enables a subsequent compensation for this effect. Therefore, images were acquired for nine blackbody temperatures (from 10 °C to 50 °C in 5 °C steps) and six climatic chamber temperatures (from 15 °C to 40 °C in 5 °C steps). The reference NUC applied in the analysis was the one calculated at a room temperature of 25 °C.

## 3. Results

### 3.1. General Functioning Validation

The basic operation of the cameras was tested and is summarized in Table 4. The acquisition software for the ST and INO IRXCAM-640 cameras provides raw data. The TE-Q1 SDK delivers processed data including thermal calibration based on the manufacturer’s data, although the details of the applied processing are not provided. 

**Figure 4:**
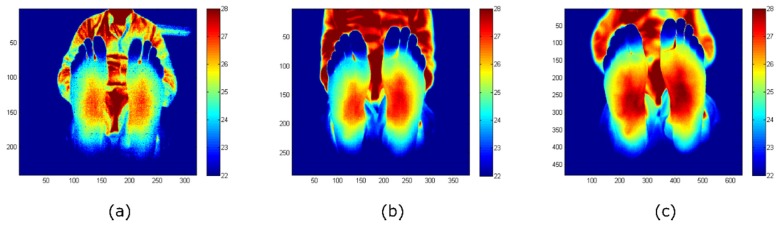
Feet images acquired with each camera: (**a**) ST; (**b**) TE-Q1; (**c**) INO IRXCAM-640. NU correction was applied to all the images to offer a clearer image. The images are color-coded indicating the temperature measurements in Celsius degrees (°C). The *x*- and *y*-axes represent the number of pixels of each sensor.

Figure 4 displays images acquired, and corrected for NUs, with each camera for the intended foot ulcer assessment. The distance between camera and object was kept in a range from 1 m to 1.2 m, aiming to focus on the sole of the foot. It must be mentioned that the detector size of the INO IRXCAM-640 camera is significantly larger than the low-cost devices. The visual inspection shows that the ST image is noisier. Noise and image resolution differences are not so evident in the images acquired with the TE-Q1 and the INO IRXCAM-640 cameras. The images also demonstrate that the depth of focus is camera-dependent. The ST image includes focused feet and background, but the latter is clearly defocused in the INO IRXCAM-640. The TE-Q1 image presents an intermediate depth of focus. These differences were expected, as the size of the optical aperture is larger in the INO IRXCAM-640 and smaller in the ST. 

### 3.2. Noise-Equivalent Temperature Difference

The measured NETDs of the tested cameras are listed in Table 5. These NETD values are coherent with the visual inspection of the images provided by the respective cameras. The INO IRXCAM-640 and TE-Q1 cameras presented similar NETD values in our tests, whereas the ST camera value was higher. For the ST and TE-Q1 cameras, the NETD values measured were higher than the ones reported by the manufacturers, being approximately 60% and 30% above nominal values, respectively.

The INO IRXCAM-640 electronics are highly configurable and include a configurable transimpedance gain in the readout circuit. The results listed were obtained using two camera configurations: the default configuration using the transimpedance gain equal to one and, an additional one, setting the transimpedance gain to its maximum value (4.5). The latter significantly improved the NETD values, since increasing the readout circuit gain increases the camera signal-to-noise ratio. 

### 3.3. Non-Uniformity Correction and Residual Non-Uniformity 

Table 6 shows the residual errors (RNU, e_NU_, and e_P-V_) after applying the multipoint NUC. The NUC performance was similar for the TE-Q1 and the INO IRXCAM-640 cameras, being the NU error levels measured below 0.1 °C as shown by the e_NU_ values. However, the ST camera exhibited worse values in our tests, below 0.2 °C, which could be explained by the higher noise level. Similarly, peak-to-valley errors (e_P-V_) were below 0.6 °C for the INO IRXCAM-640 and TE-Q1 cameras, whereas they were below 1.5 °C for the ST camera. 

### 3.4. Non-Uniformity Correction Stability and Room Temperature Dependence

**Figure 5:**
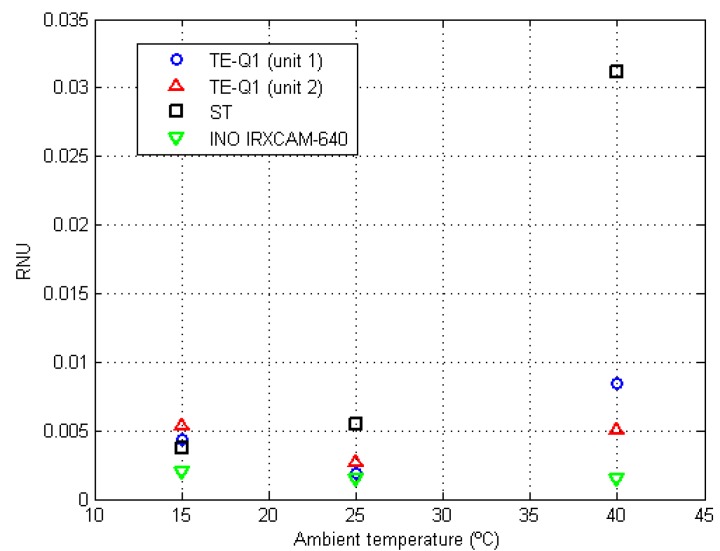
RNU values at different room temperatures (15 °C, 25 °C, and 40 °C) for the tested cameras. The symbols are color-coded, as indicated in the legend, depending on the employed camera: TE-Q1 (unit 1) blue circles, TE-Q1 (unit 2) red triangles, ST black squares, and INO IRXCAM-640 green inverted triangles.

The residual errors at room temperatures of 15 °C and 40 °C are listed in Table 7. These values, for the TE-Q1 and ST cameras, increased as compared to the values observed at 25 °C, whereas they remained relatively constant for the INO IRXCAM-640 camera. For clarification purposes, Figure 5 shows RNU variations for each tested camera at different selected ambient temperatures. Furthermore, as previously reported at 25 °C, the RNU values for the TE-Q1 cameras differed notably between units. Since they were not purchased at the same time, it might be plausible that they proceed from a different fabrication batch.

It must be noted that, although available in all the tested devices, the FPA temperature sensors were not employed in the default acquisition mode. 

### 3.5. Detector Response Curves and Stability with Varying Room Temperature

**Figure 6:**
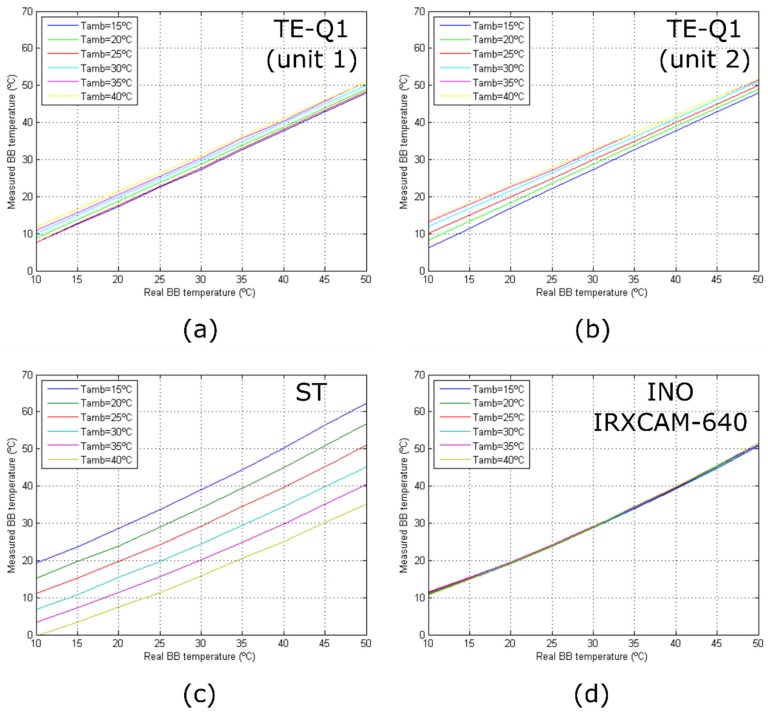
Camera response curves for different room temperatures: (**a**) TE-Q1 (unit 1); (**b**) TE-Q1 (unit 2); (**c**) ST; (**d**) INO IRXCAM-640. The curves are color-coded depending on the room temperature as seen in the legend.

Figure 6 depicts the response curves of the tested cameras at varying room temperatures. A general overview of the performance of the different cameras can be appreciated, in which the stability of the INO IRXCAM-640 is demonstrated in comparison to the other models. The TE-Q1 and ST cameras show relevant differences in the measured absolute temperatures. As mentioned above, this is not the case of the INO IRXCAM-640 camera, in which the integrated TEC used in combination with a blackbody, as an external temperature-controlled reference, provided a continuous bias correction.

Figure 6 also illustrates that only the measured responses of the TE-Q1 cameras behave linearly with temperature. This might be due to the manufacturer’s internal calibration of the microbolometer, which assumes a linear response with the sensor irradiance, as described by Planck’s law, although not with the temperature. On the other hand, the ST and INO IRXCAM-640 cameras provided raw data, and thus the manufacturer’s preprocessed step was not included. For simplicity, linearity was considered but a proper calibration is required.

## 4. Discussion

Image spatial resolution is one of the biggest traditional drawbacks of low-cost cameras when compared to high-end models. However, the actual image resolution of the selected low-cost cameras (384 × 288 pixels and 320 × 240 pixels), based on the presented work, appears to be suitable for medical applications. Specifically, the detection of anomalies in dermal thermometry, such as diabetic foot assessment, is commonly based on a contralateral limb’s temperature analysis. This procedure compares the temperature between both limbs and considers a threshold in temperature variation larger than 2.2 °C as a warning sign [17,24,28,50]. 

In our tests, the TE-Q1 and INO IRXCAM-640 cameras exhibited lower noise levels, providing comparable quality in terms of noise in their standard configuration. This improvement in the visual appearance of the images may be due to the TE-Q1 internal processing that cannot be modified or inspected by the user. In contrast, a significant noise level was observed in the ST images, which may be explained by the lack of a manufacturer’s internal calibration. 

Regarding the NETD values, the results are coherent with the initial visual inspection, showing similar levels for the TE-Q1 and INO IRXCAM-640 cameras, whereas higher levels were detected for the ST. As demonstrated, the highly configurable electronics of the INO IRXCAM-640 allows it to significantly reduce its NETD. For the intended diabetic foot application, the standard configurations of the INO IRXCAM-640 as well as the TE-Q1 cameras offer an acceptable NETD value. The ST might provide a better figure for the NETD employing a preprocessed calibration. It is worth noticing that, in the performed tests, none of the low-cost cameras reached the NETD values stated by the manufacturers.

Similarly, the NUC performance at 25 °C room temperature was similar for the TE-Q1 and the INO IRXCAM-640 cameras. However, the ST camera exhibited worse values in our tests. Therefore, since the aim was to detect a difference in temperatures around 2.2 °C, the measured NU values are considerably below this threshold for the TE-Q1 and the INO IRXCAM-640. 

The residual errors at varying room temperatures (15 °C and 40 °C) remained relatively constant for the reference camera (INO IRXCAM-640). Conversely, the values for the TE-Q1 and ST cameras increased. However, the increment was considerably higher for the ST camera, despite the internal automatic shutter that periodically acquires and applies automatic bias images. The NUC at these room temperature conditions exhibited a bad performance with peak-to-valley errors in the image higher than 4 °C in temperature, which may prevent its use in our intended medical application. Accordingly, the test demonstrates that the internal shutter of the ST camera does not correctly solve the NU degradation with room temperature variations, which can be corrected by using a TEC and an external shutter. Integrated TECs increase the price of the microbolometers and, therefore, to the best of the authors’ knowledge, they are not normally included in low-cost sensors. Alternatively, external references with controlled temperatures, such as Peltier devices, could be used in the system setup without a substantial price increment [51,52].

It is worth noticing that the residual errors observed in the TE-Q1 and INO IRXCAM-640 cameras are relatively low compared to the threshold value of 2.2 °C. The ST camera exhibited more relevant residual errors, especially the peak-to-valley error. Furthermore, the observed peak-to-valley errors in the TE-Q1 and ST cameras at high room temperatures are relevant if the cameras are used in applications based on contralateral feet temperature comparison. Therefore, the low-cost cameras require a proper calibration at high temperature limits.

Finally, the stability of the detector response with the ambient temperature was also studied showing that, as expected, the INO IRXCAM-640 combined with the blackbody bias image presents a stable response with a negligible dependence on the room temperature. Conversely, the TE-Q1 and ST exhibited errors up to 4 °C and 15 °C, respectively. Nevertheless, the temperature dependence can be modeled as a bias value proportional to the temperature. Thus, this bias can be compensated with post-processing, using a temperature sensor. Therefore, its suitability for medical applications might be fulfilled. 

The instability observed for the low-cost cameras indicates that the application of a temperature-dependent NUC correction, calculated at each room temperature, improves significantly the thermal stability of the cameras. However, this procedure is tedious, time-consuming, and not always affordable. Yet the response curves measured suggest that the stability could be significantly improved by only including a bias proportional to the ambient temperature and, thus, proportional to the FPA temperature. This action may require enough time to stabilize the FPA at the room temperature selected.

Furthermore, the TE-Q1 cameras include internal processing to provide the linear response, that is, the manufacturer’s thermal calibration is not using a simplified linear model. In the case of the cameras without this pre-processing, the calibration procedure was performed considering a linear model since only a small temperature range is used in medical applications, and, additionally, the nonlinearity of the curve does not affect the proposed tests. If a more accurate temperature measurement is required for a large temperature range in the final application, a nonlinear model could be used for the thermal calibration.

As a general recommendation based on the data presented, the thermal cameras require a thermally controlled environment for a reliable performance, particularly the low-cost models. For longitudinal tests in which the evolution in time of the absolute temperature values is measured, seasonal temperature variations might influence the acquired data. Therefore, it is recommended to select a stable room temperature as well as a camera calibration at this temperature in which the images will be acquired. Additionally, for the diabetic foot application intended, the sensor should always be operative for at least 15 min before the initial acquisition, as mentioned above. 

## 5. Conclusions and Future Work

A general overview of the capabilities of low-cost cameras, in comparison to a high-end model, was performed along with a sensor characterization. The presented data demonstrate that the performance of the TE-Q1 low-cost camera is suitable for e-health applications. The quality of the measured NETD and the RNU validated at 25 °C is comparable to the high-end INO IRXCAM-640 camera. Unfortunately, the NU of the images was degraded for extreme room temperatures such as 15 °C or 40 °C. Moreover, the response curves of the low-cost cameras also showed a significant dependence on the room temperature. Therefore, a temperature compensation algorithm should be employed in those applications in which a non-thermally controlled environment is considered, enabling the comparison of temperature measurements longitudinally.

Future work should be focused on those characteristics of the low-cost cameras exhibiting low performance which could be improved, such as the camera response (NU and measurement accuracy) at varying room temperature. The benefits of an external, temperature-homogeneous reference object in low-cost sensors should be studied in further detail. More precisely, two types of external shutters should be compared, namely an external shutter stabilized at room temperature and an external shutter thermally controlled to ensure a constant temperature. This type of temperature-controlled shutter could be implemented with a low-cost setup, for example, based on a Peltier device, and compared in performance with an external shutter based on a blackbody.

## Figures and Tables

**Table 1:** Characteristics of low-cost commercially available long-wavelength infrared (LWIR) cameras as provided by their manufacturers.

	Seek Thermal CompactPRO (ST)	Thermal Expert TE-Q1 Plus (TE-Q1)	Opgal Therm-App	Flir ONE PRO
Detector size	320 × 240 pixels	384 × 288 pixels	384 × 288 pixels	160 × 120 pixels
Frame rate	>15 Hz	<9 Hz	<9 Hz	8.7 Hz
NETD/MRTD ^1^	NETD < 70 mK	NETD < 50 mK	NETD < 70 mK	MRTD = 150 mK
Phone compatibility	Android/iOS	Android	Android	Android/iOS
Manufacturer	Seek Thermal Inc. (Santa Barbara, CA, USA)	I3system, Inc. (Daejeon, Republic of Korea)	Opgal Optronic Industries Ltd. (Karmiel, Israel)	FLIR Systems, Inc. (Wilsonville, OR, USA)

^1^ NETD, noise-equivalent temperature difference; MRTD, minimum resolvable temperature difference.

**Table 2:** Characteristics of the INO IRXCAM-640 [30].

	INO IRXCAM-640
Detector size	640 × 480 pixels (pixel pitch: 25 μm)
Frame rate	30 Hz (1 channel)/60 Hz (2 channels)
NETD	NETD <50 mK (ULIS-04-17-1, ULIS measurements with 300K target and f/1 lens)
Size (W × H × D)	65 mm (H) × 59 mm (W) × 125 mm (L)
Data interface	Gigabit Ethernet (GigE) Link/CVBS (NTSC/PAL)
Other characteristics	Integrated thermoelectric cooler (TEC) Configurable transimpedance gain in the ROIC

**Table 3:** NETD test characteristics.

	TE-Q1	ST	INO IRXCAM-640
Blackbody temperature	300 K	300 K	300 K
Room temperature	25 °C (stabilized)	25 °C (stabilized)	25 °C (stabilized)
NETD measurement method	Spatial NETD + quantification effect processing	Spatial NETD	Spatial NETD
Lens f-number (manufacturer data)	f/1.0	not provided	f/0.86

**Table 4:** Summary of camera characteristics according to preliminary tests.

	TE-Q1	ST ^1^	INO IRXCAM-640
Image acquisition	LINUX/Windows/SDK provided by the manufacturer	LINUX/Windows Libseek-thermal driver	Windows GUI and protocol provided by the manufacturer
Image format	16-bit/float	16-bit	16-bit raw data
Raw/processed pixel data	Image includes processing	Raw data	Raw data

^1^ Seek camera SDK for Linux/Windows exists but it was not available at the time of the tests.

**Table 5:** NETD results at T_amb_ = 25 °C and blackbody temperature T_obj_ = 27 °C.

	TE-Q1 (unit 1)	TE-Q1 (unit 2)	ST	INO IRXCAM-640 (Gain = 1)	INO IRXCAM-640 (Gain = 4.5)
NETD	64.4 mK	63.3 mK	110.8 mK	63.4 mK	48.3 mK

**Table 6:** Residual non-uniformity (RNU), e_NU_, and e_P-V_ values at T_amb_ = 25 °C and blackbody temperature T_obj_ = 32 °C.

	TE-Q1 (unit 1)	TE-Q1 (unit 2)	ST	INO IRXCAM-640
RNU	0.0019	0.0027	0.0055	0.0015
e_NU_ (°C)	0.059	0.082	0.172	0.047
e_P-V_ (°C)	0.473	0.593	1.481	0.394

**Table 7:** RNU, e_NU_, and e_P-V_ values for a blackbody temperature T_obj_ = 32 °C and different room temperatures.

T_amb_		TE-Q1 (unit 1)	TE-Q1 (unit 2)	ST	INO IRXCAM-640
15 °C	RNU	0.0044	0.0054	0.0037	0.0020
	e_NU_ (°C)	0.126	0.151	0.153	0.061
	e_P-V_ (°C)	0.762	1.019	1.212	0.517
40 °C	RNU	0.0084	0.0051	0.0312	0.0015
	e_NU_ (°C)	0.281	0.173	0.554	0.045
	e_P-V_ (°C)	1.906	1.620	4.098	0.382
